# Factors Contributing to Urban Malaria Transmission in Sub-Saharan Africa: A Systematic Review

**DOI:** 10.1155/2012/819563

**Published:** 2012-10-18

**Authors:** Prathiba M. De Silva, John M. Marshall

**Affiliations:** ^1^Department of Medicine, Imperial College London, London W2 1PG, UK; ^2^MRC Centre for Outbreak Analysis & Modelling, Department of Infectious Disease Epidemiology, Imperial College London, London W2 1PG, UK

## Abstract

Sub-Saharan Africa suffers by far the greatest malaria burden worldwide and is currently undergoing a profound demographic change, with a growing proportion of its population moving to urban areas. Urbanisation is generally expected to reduce malaria transmission; however the disease still persists in African cities, in some cases at higher levels than in nearby rural areas. *Objective*. This paper aims to collate and analyse risk factors for urban malaria transmission throughout sub-Saharan Africa and to discuss their implications for control. *Methods*. A systematic search on malaria and urbanisation was carried out focusing on sub-Saharan Africa. Particular interest was taken in vector breeding sites in urban and periurban areas. *Results*. A variety of urban vector breeding sites were catalogued, the majority of which were artificial, including urban agriculture, tyre tracks, and ditches. Natural breeding sites varied according to location. Low socioeconomic status was a significant risk factor for malaria, often present in peri-urban areas. A worrying trend was seen in the adaptation of malaria vector species to the urban environment. Urban malaria is highly focused and control programs should reflect this. *Conclusion*. As urbanisation continues and vector species adapt, continued monitoring and control of urban malaria in sub-Saharan Africa is essential.

## 1. Background

Despite recent declines in *Plasmodium falciparum* malaria transmission, largely due to increased distribution of long-lasting insecticide-treated nets (LLINs) and a switch to artemisinin-based combination therapy (ACT) drugs, sub-Saharan Africa still suffers greatly from the disease. According to World Health Organization (WHO) estimates, in 2010, of the 655,000 deaths attributed to malaria worldwide, 91% of these were in Africa [1]. At the same time, Africa's demography is rapidly changing, with an increasing number of people moving to urban areas. In West Africa, the population growth rate for urban areas is estimated at 6.3%, which is more than double the total population growth rate [2], and it is predicted that, by 2035, the urban population of sub-Saharan Africa will outnumber the rural one [3]. As Africa becomes increasingly urbanized, factors contributing to urban malaria will become more relevant.

The general consensus is that urbanization will lead to decreased malaria transmission. One recent modelling study predicts a 53.5% reduction in malaria transmission by 2030, largely due to expected demographic changes [4]. It is thought that urbanization leads to improved infrastructure, better-quality “mosquito-proof” housing, increased access to healthcare, and a reduction in vector breeding sites. Malaria vector species are known to prefer clean water for breeding, which is difficult to come by in polluted urban areas, and the higher ratio of humans to mosquitoes is also thought to lead to a decreased human biting rate [5].

However, despite these encouraging factors, malaria transmission persists in African cities and, in some cases, at even higher levels than in surrounding areas [6]. Indeed, there are African cities experiencing entomological inoculation rates (EIRs) greater than 80 infective bites per person per year [7]. A variety of factors may contribute to this, including socioeconomic status, urban agricultural practices and poorly-monitored land use [8]. Uncontrolled urban expansion can lead to increased malaria transmission as town planners are unable to keep up with sprawling city boundaries and rural practices, which are conducive to vector breeding sites and incorporated into the urban fringes. Furthermore, areas of low socioeconomic status, often at the periphery of cities, are at particular risk. Here, poor-quality housing, unpaved roads, and reduced access to healthcare provide little protection against the disease [9].

A number of systematic reviews have investigated the impact of urbanization on malaria transmission in sub-Saharan Africa [10–12], dividing transmission into urban, periurban, and rural settings. Annual EIRs compiled across dozens of African cities show a strong tendency for transmission to increase in a gradient from urban to periurban to rural areas—in the most recent meta-analysis, the average EIRs were 18.8, 63.9, and 126.3 infective bites per year, respectively [10]. However, urban malaria transmission varies according to a number of additional factors such as location (e.g., altitude, proximity to a sea, river, or floodplain), climate, land use, human movement patterns, socioeconomic factors, local vector species, vector breeding sites, waste management, and local malaria intervention programs. This paper aims to identify the important factors in urban malaria transmission in sub-Saharan Africa, to better understand their interactions, and to discuss their relevance to policy makers in an increasingly urbanized continent.

## 2. Methods

### 2.1. Literature Search

A systematic search on the impact of urbanisation on malaria transmission in sub-Saharan Africa was carried out in April 2012 by the first named author on the following electronic databases: EMBASE, HMIC, Medline, Maternity and Infant Care, Psycinfo, and Transport through the OVIDSP gateway from 1946 to 2012. The search was performed as follows: [(urban) OR (urbanisation) OR (urbanization)] AND [(malaria) OR (*Plasmodium*) OR (*Anopheles*)]. 

“Africa” was originally included as a key word, but was subsequently left out as it resulted in several papers being neglected that referred to specific African countries rather than the African continent. Papers referring to Africa were therefore chosen manually.

### 2.2. Inclusion Criteria

**Figure 1:**
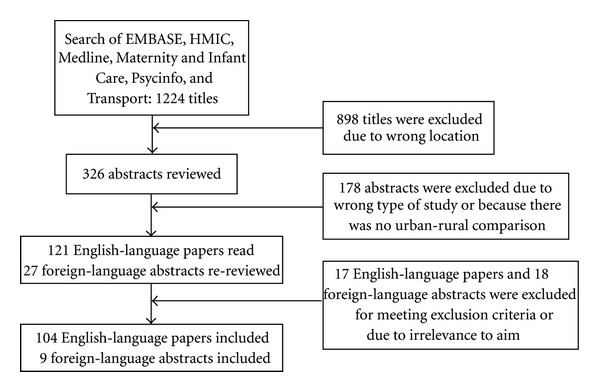
Flow chart of study selection process.

The above key words yielded a total of 1,224 published articles. The authors agreed that the articles included from the search should meet the following criteria: (i) description of malaria burden/transmission/control in urban settings; (ii) study location in sub-Saharan Africa; (iii) English-language abstract. The first named author scanned all articles by title, eliminating those that did not concern sub-Saharan Africa, leaving 326 abstracts to be read. A further 178 abstracts were rejected that did not meet the inclusion criteria. Full texts of the remaining 148 articles were read, unless in a foreign language, in which case the abstracts were read again and studies were excluded if they did not meet the inclusion criteria upon further reading. Where relevance to the inclusion criteria was questioned, the second named author independently evaluated the article and consensus was quickly reached. Finally, 104 English-language articles and nine foreign-language abstracts were identified as relevant to the topic. The literature selection process is summarized in Figure 1.

## 3. Results of Literature Search

Relevant papers dated back to 1984. Supplementary Table 1 (see Supplementary Material available on line at doi:10.1155/2012/819563) shows the results of the search and the location, publication year, and topics addressed for each study. The authors agreed on which topics to focus on before coding them for each paper. As this paper is largely qualitative, points of interest were noted for each paper and collated for comparative analyses. The number of papers that contributed information to each topic is summarized in Table 1, along with the location and year of publication. We found that a comparable number of studies addressed the role of the vector breeding sites in urban malaria transmission (*n* = 51) as those addressing the role of geographic disparities (*n* = 48). For papers referring to vector breeding sites, we recorded the number of studies referring to specific sites and summarized these in Table 2. Many papers were synonymous in their findings, so were noted but not necessarily included in the discussion.

## 4. Discussion

### 4.1. Urban, Periurban and Rural Transmission

As mentioned in the background, dozens of African cities show a clear trend of increasing malaria transmission from urban to periurban to rural settings [10–12]. For example, in Ouagadougou, Burkina Faso, the *P. falciparum* parasite rate (PfPR) has been estimated at 24.1% in the urban center, 38.6% in its periurban surroundings, and 68.7% in neighboring rural areas [13]. This is largely due to the fact that African cities tend to grow outwards with perimeters consisting of relatively underdeveloped, poorly serviced settlements [14]. Recent migrants from rural areas tend to bring their rural practices with them, creating a multitude of vector breeding sites [15], and poor quality housing provides less protection against mosquito bites [16]. 

However, it should be noted that this is not a universal trend. In Libreville, Gabon, malaria transmission was found to be the highest in the urban center (EIR of 87.9 infective bites per person per year) and the lowest in the periurban surroundings (EIR of 13.3 per person per year) as a consequence of slum-like conditions in the urban center being surrounded by more affluent periurban suburbs [7]. In Cotonou, Benin, malaria prevalence was highest in an intermediate zone (PfPR among children aged 6–12 of 9.0%) between the urban center (PfPR of 2.6%) and periphery (PfPR of 2.5%). This has been explained by the abundance of urban agriculture in the intermediate zone and a salty lagoon at the periphery making it less conducive to the primary malaria vector *Anopheles gambiae* [17]. This shows that we should not confine our impression of urban malaria simply to urban centres, but we should also base it on an understanding of the underlying geography.

### 4.2. Malaria Vectors

Malaria in humans results from infection by any of five species of *Plasmodium* transmitted by approximately 50 species of mosquitoes, all belonging to the genus *Anopheles*. In sub-Saharan Africa, the majority of deaths are caused by *P. falciparum* and transmitted by *An. gambiae* s.s. and its close relative *Anopheles arabiensis*. These species are part of a larger species complex, *An. gambiae* s.l., of which *Anopheles melas* is also a member [18]. *An. gambiae* s.s. can further be divided into M and S molecular forms. The M form is better adapted to urban and dry environments and tends to reproduce alongside irrigated fields and permanent or semipermanent swamps. The S form is better adapted to rural and humid forest areas and prefers temporary pools and brick-made ravines [7, 15, 19, 20]. *An. melas* contributes to coastal malaria and is usually found in salt water lagoons [21]. Another vector species, *Anopheles funestus*, also contributes to malaria transmission on the continent and thrives in dry and periurban environments [22]; *Anopheles moucheti,* a rare vector species, breeds in slow-moving rivers [23]. In a recent study in urban Libreville, Gabon [7], *An. gambiae* s.s. S form accounted for 99.5% of all vectors collected, while the M form accounted for 0.2% and *An. melas* accounted for 0.3%. Interestingly, all collected species and sub-species of the *An. gambiae* s.l. complex were positive for malaria sporozoites. 

Urban environments are less favourable for vector species, particularly *An. gambiae*, which has a strong preference for unpolluted waters [5]. The lifespan of *An. gambiae* in urban areas was measured to be less than half its lifespan in rural areas (4.1 versus 11 days) in a study in Kinshasa, Democratic Republic of the Congo [24]. Mosquito dispersal is also much more limited in urban areas due to the higher housing density [25], causing urban malaria transmission to be highly focal [18].

### 4.3. Natural Vector Breeding Sites and Environmental Factors

The heavy burden of malaria in rural Africa is testimony to the ability of natural breeding sites to sustain vector populations. Natural breeding sites, although less common in urban areas, are nevertheless present. Field studies suggest that anopheline larvae are most likely to be found in permanent, shallow, sunlit pools of water of perimeter greater than 10 m [26–28]. Temporary pools are less favoured because they may not provide sufficient time for eggs to develop and emerge as adults. It has also been suggested that they are more likely to be disturbed by human activity [26–28]. A high groundwater table is particularly conducive to breeding sites as the absence of surface runoff allows pools of stagnant water to develop [29]. Of the natural vector breeding sites referred to in the literature search, the most common were ponds (*n* = 8) and swamps (*n* = 13). Also mentioned were seepages, springs, and streams and, in one study, *An. gambiae* were discovered in over 100 trees, suggesting tree holes as a favoured ovipositing site [30]. 

#### 4.3.1. Coastal Environments

Malaria in coastal African cities has been partially attributed to the colonization of shallow salt waters by *An. Melas—*a less efficient, salt-water-breeding vector species [17, 31]. Clay soils of lagoons have also been noted for collecting stagnant water, providing excellent aquatic conditions for vectors species, with studies in Cote d'Ivoire and Tanzania documenting strong correlations between the presence of clay soil and anopheline mosquitoes [26, 29].

#### 4.3.2. Rivers and Floodplains

Rivers and their floodplains provide great breeding grounds for mosquitoes in riverside urban communities, as demonstrated by the strong association between malaria risk and proximity to a floodplain. Large fields with loamy/clay soils tend to collect stagnant water from rivers and provide optimal conditions for anopheline breeding [32]. In Adama, Ethiopia, for example, households within 250 m of a floodplain have been shown to have a 22 times higher risk of contracting malaria than households further than 950 m away [33]. Sometimes it is the human activity associated with a setting that creates fertile conditions for vector breeding. For example, farms around the confluences of the Blue and White Nile in Khartoum, Sudan, are foci of malaria transmission, as are irrigated rice fields in Dioro, Mali, alongside the Niger River [34].

#### 4.3.3. Altitude

Altitude is commonly thought to play an important role in limiting malaria in the tropical highlands by negatively influencing the development of vector species. In a study of malaria prevalence in south-western Uganda, altitudes higher than 1,500 m were shown to be associated with low malaria risk [35]; however, the presence of vector species at these altitudes cannot be ruled out since a study in the Kenyan highlands revealed high densities of *An. gambiae* mosquitoes in a town 1,650 m above sea level and still more at altitudes higher than 2,000 m [30].

### 4.4. Artificial Vector Breeding Sites

It is widely regarded that artificial rather than natural vector breeding sites provide the most abundant sources of mosquito larvae in African urban centres [32, 36, 37]. This is reflected in Table 2, which shows that artificial vector breeding sites were referred to almost three times more than natural sites in this systematic review. Citation numbers are not conclusive evidence for such a comparison; however analysis of the papers from which these numbers were drawn (Supplementary Table 1) does not suggest any obvious bias. Urban agriculture was the most cited breeding site in the literature search (*n* = 36), followed by drains/gutters (*n* = 9), ditches (*n* = 8), tyre tracks (*n* = 8), and water pipes (*n* = 6). Also mentioned were water tanks, construction sites, and swimming pools. Some of these sites, such as tyre tracks and swimming pools, were found to contain all life stages of *An. gambiae*, suggesting that they were particularly productive habitats [26, 38] and were found mainly in poorly-drained, periurban areas [39].

#### 4.4.1. Urban Agriculture

Over the last decade, urban agriculture has become commonplace in sub-Saharan Africa, expanding into the peripheral belts and centres of many towns and cities [15]. Its benefit is that it increases food security while combating malnutrition and poverty; however, it also provides optimal conditions for vector breeding, leading to a higher risk of malaria transmission in its vicinity [36, 40]. Agricultural trenches create ideal breeding sites due to the formation of shallow water between seed beds and, in one study in Abidjan, Cote d'Ivoire, anopheline larvae were present in over half [26]. In another study in Cote d'Ivoire, rice fields were found to have the highest likelihood of anopheline presence throughout both wet and dry seasons [6]. Other breeding sites include irrigation wells, noncemented wells, ditches for furrow systems, and human footprints [29, 41–43]. Larger breeding sites are more productive as they are less likely to be disturbed by irrigation.

Higher mosquito densities naturally lead to elevated levels of malaria transmission for people who either work on or live near urban agricultural fields [15, 40, 44]. For example, in a study in Maputo City, Mozambique, malaria parasitaemia was found to be higher among those who worked in urban agricultural areas throughout the city, irrespective of other factors such as urban or periurban location [45]. Urban agriculture is often associated with socioeconomic advantages, such as piped water, refuse collection, a sewage system, and better education; however, data from Accra, Ghana, suggests that the increase in vector breeding sites is sufficient to counteract these beneficial effects in terms of malaria transmission [8]. There are currently no known initiatives in place for controlling malaria associated with urban agriculture, and control here should be mindful of socioeconomic considerations.

#### 4.4.2. Drains, Ditches, and Gutters

While agriculture provides the most productive urban vector breeding sites, drains and ditches may provide more common habitats. In a study in Dar es Salaam, Tanzania, there were three times more anopheline-positive drains and ditches compared to agricultural breeding sites, and anopheline presence was much more likely in drains that were blocked [32]. Blockages are often due to poor sanitation and lead to reduced water flow and accumulation of stagnant water pools which are ideal for mosquito breeding. Gutters provide a similar breeding site for mosquitoes in both the wet and dry seasons and were specifically noted by a recent study in Abeokuta, Nigeria [46].

#### 4.4.3. Tyre Tracks

Tyre tracks were the second most-cited artificial vector breeding site. In Malindi, Kenya, they accounted for as much as 29% of all water bodies that were positive for mosquitoes [38]. Tyre tracks are more common in areas of high socioeconomic status, which tend to house more vehicle owners while still having roads of sufficiently poor quality to lead to the formation of potholes, tyre tracks, and other artificial breeding sites. 

#### 4.4.4. Swimming Pools

In another study in Malindi, unused swimming pools were found to provide a particularly productive habitat for *Anopheles* immature stages [47]. Of the 250 habitats identified in the study, 66 were swimming pools, and these were found to have the highest abundance of *Anopheles* mosquitoes. Hotel workers, tourists, and domestic workers may be at heightened risk of malaria transmission in areas with an abundance of unused pools.

#### 4.4.5. Water Pipes

Water pipes can lead to breeding site formation in a variety of ways, most frequently when they are broken and pools of water collect [5]. Pipes often break as a result of poor installation or quality, clay soil expansion and contraction, construction work, and as an opportunity to procure free water for sale or consumption [48]. Water sources that are further away from pipes are more likely to be anopheline positive because water flow from nearby pipes may disturb the water surface, reducing the breeding site quality [49]. Artificial water storage containers can also serve as breeding sites, and car washing has been found to provide excellent habitats for larval development [39].

### 4.5. Human Factors

#### 4.5.1. Socio-Economic Status

Higher socioeconomic status is associated with a number of factors that lead to reduced malaria transmission, from piped water and better refuse collection to better education, higher exposure to TV and radio prevention campaigns, and increased ability to afford prevention methods and treatment [50–52]. These factors contribute to a better awareness of vector breeding sites, malaria transmission, and control among people of higher socioeconomic status. The higher socioeconomic status of urban dwellers is a major factor contributing to their reduced risk of contracting malaria [53]; within cities, socioeconomic factors contribute to increased transmission in poorer areas with slum-like conditions, as seen in Libreville, Gabon [7], and in the periurban areas of many cities. 

#### 4.5.2. Household Factors

Better-quality housing decreases the risk of malaria as it minimizes entry points for mosquitoes during the night. To illustrate this, a study in Gambia showed that houses with malaria-infected children are more likely to have mud walls, open eaves, and absent ceilings than those with uninfected children [16]. Floors comprised of earth bricks are also associated with lower malaria risk as inhabitants are more likely to sleep on raised beds to avoid ground moisture, in turn eluding bites from *An. gambiae* mosquitoes which search for blood close to the ground [16]. Interestingly, a study in Burkina Faso found that electricity use was associated with increased malaria risk, as the alternative of biomass fuel burning produces smoke that is thought to deter mosquitoes from entering houses [54]; however, electricity use in better-quality housing would presumably not show this trend.

#### 4.5.3. Community Factors

Hygiene, sanitation, and waste collection are key determinants of malaria transmission which, while household responsibilities, have a community-level effect on disease transmission. As an example, the more the households dispose of waste properly, the lower the risk of liquid waste collecting in pools of stagnant water and forming vector breeding sites. In Accra, Ghana, being connected to a toilet was found to be even more important than waste removal in reducing community malaria mortality [55]; however, toilets are also potential areas of mosquito activity, and septic tanks within communities are a potential source of vector breeding sites [56].

#### 4.5.4. Travel

The flipside of lower malaria prevalence in urban areas is that immunity is also reduced, making urban dwellers more susceptible to the disease upon exposure. Reduced immunity in urban populations means that, when urban residents travel to rural areas, they are at risk of contracting serious cases of malaria [57]. Due to their reduced immunity, city dwellers are more likely to contract malaria both when they travel to rural areas and when malaria-infected individuals travel to the city. This is supported by studies of urban populations in Burkina Faso, Cote d'Ivoire, and Zambia, all of which reveal a strong association between malaria infection and a recent trip to a rural area [58–60]. Furthermore, in West African cities, heightened EIRs have been observed in October, which is a time when urban dwellers return from their summer vacations in rural areas and rural youths travel to cities in search of work following the rural agricultural season [34].

### 4.6. Vector Factors (Adaptation and Mutualism)

*An. gambiae *is demonstrating a worrying trend of adaptation to polluted waters in urban environments [5]. In recent years, the species has been found breeding in highly polluted water sources in Cote d'Ivoire [6] and Cameroon [19], and in water-filled domestic containers in Accra, Ghana [61]. In Lagos, Nigeria, and Kisumu and Malindi, Kenya, *An. gambiae* s.s. larvae have been found in water sources with high concentrations of heavy metals such as iron, copper, and lead, and other contaminants such as human faeces and petrol [52, 62]. *An. arabiensis*, although tolerant of turbidity, was less tolerant of these pollutants [62], as was *An. funestus *[63], suggesting that these species are less adapted to polluted water sources than *An. gambiae* s.s. These findings suggest that the pollution associated with urbanisation will not necessarily continue to reduce vector densities in African cities, and urban vector control will become increasingly relevant in years to come. Furthermore, the widespread use of ITNs and IRS, combined with insecticide usage in agriculture, is posing a strong selective pressure on vector populations to develop insecticide resistance, suggesting that future IVM programs will need to rely on a wide range of vector control strategies [5].

The mutually beneficial relationship between *Culex quinquefasciatus*—a nonmalaria vector—and *An. gambiae* can lead to elevated malaria vector densities in urban environments [6, 32]. *C. quinquefasciatus* breeds very efficiently in artificial sites like drainage facilities and, once inhabiting these sites, creates an environment in which *An. gambiae* can also breed. How this happens is yet to be explored. In a study in Abeokuta, Nigeria, *An. gambiae* were discovered in gutters blocked by refuse and sewage, but only after they had already been inhabited by *Culex* species [46].

### 4.7. Implications for Control

The current approach of the WHO to control malaria in sub-Saharan Africa is a combination of vector control, in the form of LLINs and indoor residual spraying with insecticides (IRS), and the distribution of ACT drugs for treatment [64]. Insecticide-treated nets (ITNs) have been shown to be highly efficient at reducing malaria on a community level in urban Ghana [65]; other interventions, such as larviciding and removal of vector breeding sites, are appropriate in both urban and periurban settings. Improved housing, for instance, by using corrugated iron instead of thatched roofing [66], reduces entry points for mosquitoes and is appropriate in less affluent urban settings.

Malaria transmission in urban and periurban areas is highly focused around vector breeding sites, which tend to be more numerous in areas of lower socioeconomic status. Control strategies should therefore adopt an element of spatial targeting rather than targeting a wide urban area uniformly. Vector breeding sites are common in areas with slum-like conditions [7] and in areas where urban agriculture is practiced [36, 40]. Here, emphasis should be placed on both removal of breeding sites and protective measures for the local population. One area where control could be improved is urban agriculture, as there are currently no known initiatives in place that deal with urban agriculture-associated malaria specifically. That said, we must be careful not to hinder the socioeconomic benefits of urban agriculture, such as better education and piped water. Provision of toilets may help to remove some breeding sites [55]. Communities of low socioeconomic status are less likely to be able to afford protective measures such as LLINs and IRS and treatments such as ACTs, so distribution programs and education campaigns should be targeted at these communities [35, 67]. Control strategies should also target urban environments conducive to natural breeding sites, such as coastal lagoons, rivers, and floodplains [26, 29, 32–34].

Sites known to be conducive to vector breeding—such as agricultural fields, tyre tracks, ditches, swimming pools, and construction sites—should be targeted for control. Larviciding should be prioritised since larvae contained within aquatic sites are easier to control than free-flying adults [6], and its annual cost per individual is less than two-thirds that of ITNs [68]. In Dar es Salaam, Tanzania, larviciding has contributed to reported reductions in malaria transmission of up to 87% [68]. According to the WHO Global Malaria Programme, larviciding should be included as an additional measure to IRS and LLINs, especially in urban areas, where it is cheaper and easier to larvicide the limited urban breeding sites than to distribute nets and apply insecticide to numerous households [69]. Chemical treatment of swimming pools [70] and unclogging and treating stagnant drains will reduce larval densities and decrease larviciding costs even further [32]. Integrated vector management (IVM) provides the WHO's decision-making framework for vector control, and its emphasis on local evidence and participation makes it an ideal framework for effectively utilising a community's resources to control the evolving phenomena of urban malaria [71].

## 5. Conclusion

The studies selected for this paper provide a well-rounded picture of the range of factors that contribute to malaria transmission in urban sub-Saharan Africa. Clearly, there is great variation from city to city and from town to town depending on a myriad of environmental, ecological, and socioeconomic factors. However, from a holistic analysis, it is clear that there are patterns of malaria transmission, an understanding of which will help to inform the development of future urban malaria control programs. 

**Figure 2:**
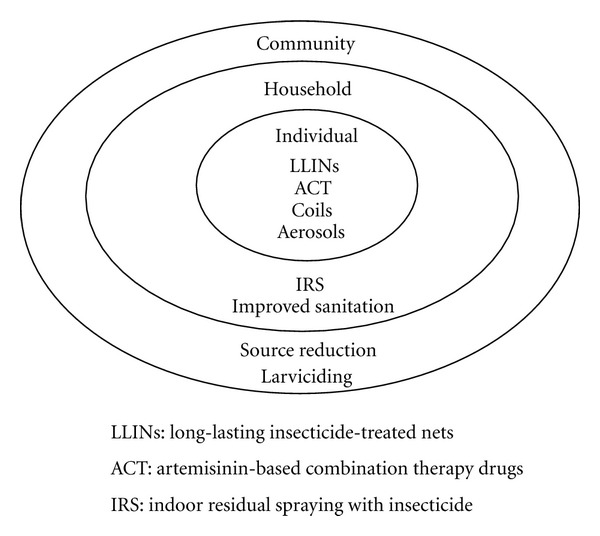
Interventions against urban malaria at the community, household, and individual level.

In terms of priorities, urban malaria is most efficiently controlled through highly focused, community-level interventions. The emphasis here should be on eliminating vector breeding sites through larviciding and other measures. While LLINs and IRS are the gold standard for vector control in rural areas, there is much greater potential to identify and eliminate breeding sites in urban settings. Attention should be paid to both natural and artificial breeding sites, as summarized here. That said, individual and household-level interventions—for example, LLINs, ACTs, improved sanitation, and IRS—should continue to be strongly encouraged (Figure 2).

The role of monitoring and targeting should be emphasized, as urban malaria is known to be highly focused. These activities should form the basis of an effective IVM program. Predictable areas of high transmission should be closely monitored, including areas of low socioeconomic status, which are often located in periurban areas and are more likely to house vector breeding sites but less likely to have protective measures against vectors and malaria. Another area where close attention should be paid is near urban agricultural fields and environmentally susceptible sites such as coastal lagoons, rivers, and floodplains where human activity can enhance the suitability for breeding sites.

As urbanization continues and malaria vectors continue to adapt to the urban environment, the considerations in this paper will become increasingly relevant. We argue for the continued monitoring of urban malaria, to determine foci of transmission and interventions appropriate to these and other urban areas. We support the continuation of IVM programs, which should be tailored to each individual area, as a growing proportion of sub-Saharan Africa's population represents this demographic.

## Supplementary Material

The Supplementary Material consists of an Excel spreadsheet outlining all papers analysed for the systematic review. It details the location of study, year of publication and topics addressed for each paper returned by the search.

## Figures and Tables

**Table 1:** Summary of results by year, location, and number of citations.

Topic	Years published	Locations	No. citations
Urban, peri-urban, rural comparisons	1986–2012	Senegal, Gabon, Kenya, Congo, Mozambique, Ethiopia, Uganda, Cameroon, Tanzania, Burkina Faso, Nigeria, Angola, Ghana, Cote d'Ivoire, Benin, Niger, Djibouti, Dakar, Sudan, DRC, Zambia, and Madagascar	48
Vector factors	1987–2012	Benin, Gabon, Kenya, Tanzania, Senegal, Sudan, Nigeria, Uganda, Ghana, Gambia, and DRC	18
River	1997–2012	Gambia, Mali, Tanzania, Sudan, Cameroon, and Niger	6
Coast	1992–2012	Cote d'Ivoire, Benin	5
Altitude	1993–2012	Tanzania, Uganda, Kenya, and Cameroon	5
Vector breeding sites (artificial & natural)	1986–2012	Mali, Mozambique, Ethiopia, Benin, Cote d'Ivoire, Senegal, Tanzania, Burkina Faso, Kenya, Ghana, Angola, Uganda, Nigeria, Sudan, Cameroon, Gambia, DRC, and Gambia	51
Socio-economic status	1990–2012	Kenya, Tanzania, Ghana, Angola, Nigeria, Malawi, Burkina Faso, and Gambia	12
Household	1993–2011	Ghana, Gabon, Burkina Faso, Tanzania, Cote d'Ivoire, and Gambia	9
Community	2010–2011	Ghana, Gabon	2
Travel	1994–2012	Gabon, Kenya, Guinea, Burkina Faso, Cote d'Ivoire, and Gambia	6
Adaptation/mutualism	2005–2011	Gabon, Kenya, Cameroon, Benin, Burkina Faso, Tanzania, Senegal, Ghana, Nigeria, and Cote d'Ivoire	13
Control	1984–2012	Kenya, Mozambique, Ghana, Tanzania, Angola, Burkina Faso, Uganda, Malawi, Gambia, DRC, and Cote d'Ivoire	22

**Table 2:** Urban vector breeding sites by number of citations.

	Vector breeding site	Number of studies
Natural	Swamps	13
	Ponds	8
	Puddles	7
	Marshes	4
	Streams	4
	Seepages	3
	Springs	1
	Lakes	1
	Tree holes	1

Total		42

Artificial	Urban agriculture	36
	Drains/gutters	9
	Ditches	8
	Tyre tracks	8
	Pipes	6
	Domestic containers	5
	Water tanks/reservoirs	5
	Construction	4
	Swimming pools	3
	Canal	3
	Foundations	2
	Septic tanks	2
	Tyres	2
	Bathtubs	1
	Dam	1

Total		95
